# The impact of sleep apnea syndrome on the altered lipid metabolism and the redox balance

**DOI:** 10.1186/s12944-021-01604-8

**Published:** 2021-12-05

**Authors:** Branislav Kollar, Pavel Siarnik, Alzbeta Hluchanova, Katarina Klobucnikova, Imrich Mucska, Peter Turcani, Zuzana Paduchova, Barbora Katrencikova, Maria Janubova, Katarina Konarikova, Lubica Argalasova, Stanislav Oravec, Ingrid Zitnanova

**Affiliations:** 1 1st Department of Neurology, Faculty of Medicine, Comenius University, Bratislava, Slovakia; 2 Department of Neurology, University Hospital Bratislava, Bratislava, Slovakia; 3 Outpatient Clinic for Sleep-Disordered Breathing, University Hospital Bratislava, Bratislava, Slovakia; 4 Institute of Medical Chemistry, Biochemistry and Clinical Biochemistry, Faculty of Medicine, Comenius University, Bratislava, Slovakia; 5 Institute of Hygiene, Faculty of Medicine, Comenius University, Bratislava, Slovakia; 6 1st Department of Internal Medicine, Faculty of Medicine, Comenius University, Bratislava, Slovakia

**Keywords:** Obstructive sleep apnea (OSA), Dyslipoproteinemia, LDL subfractions, HDLsubfractions, Lipoperoxides

## Abstract

**Background:**

Obstructive sleep apnea (OSA) is a disorder with a significant risk for cardiovascular diseases. Dyslipidemia and redox imbalance belong to potential mechanisms linking OSA with the development of vascular diseases. The main aim of this study was the evaluation of the presence of lipid abnormalities in OSA patients, focusing on small dense low-density lipoprotein (LDL) and high-density lipoprotein (HDL) subfractions and determination of the redox imbalance by evaluating the marker of oxidative damage to plasma lipids - lipoperoxides.

**Methods:**

The study included 15 male subjects with polysomnographically confirmed OSA and 16 male healthy controls. Plasma levels of total cholesterol, LDL and HDL and their subfractions, triacylglycerols and lipoperoxides were determined in all study individuals. Plasma LDL and HDL subfractions were separated by the Lipoprint system which is a polyacrylamide gel electrophoresis. Lipoperoxide levels were determined spectrophotometrically.

**Results:**

OSA patients had significantly higher triacylglycerols, total cholesterol and LDL-cholesterol compared to healthy controls. HDL cholesterol was not significantly different. Of the LDL and HDL subfractions, OSA patients had significantly lower levels of atheroprotective LDL1 and large HDL subfractions and significantly higher levels of atherogenic small dense LDL3–7 and HDL8–10 subfractions. Lipoperoxide levels in patients with OSA were significantly elevated compared to healthy individuals.

**Conclusion:**

The lipoprotein pro-atherogenic phenotype was found in individuals with OSA characterized by increased levels of atherogenic lipoprotein subfractions and reduced levels of atheroprotective subfractions. In addition, a plasma redox imbalance was found in patients with OSA compared to controls by detecting higher oxidative damage to lipids. Abnormalities in lipoprotein levels in patients with OSA, as well as the redox imbalance, could lead to an acceleration of the atherosclerotic process in predisposed individuals and thus represent a significant risk factor for vasular diseases.

## Background

One of the most common sleep disorders characterized by an increased risk of vascular disease is obstructive sleep apnea (OSA) [1]. It is a disorder characterized by episodes of breathing interuption during sleep, leading to chronic intermittent hypoxia and sleep fragmentation by numerous awakening periods [2]. In Europe, the disease affects more than 20% of the middle-aged adult population and the prevalence even exceeds 50% in some countries [3]. To date, it has not been clearly explained which pathophysiological mechanisms of OSA lead to the development of vascular diseases. Elevation of the atherogenic index of plasma was observed in the OSA population and it was related to the disease severity [4].

From previously published works there is known the association between intermittent hypoxia and a wide range of pathological processes, such as endothelial dysfunction, activation of the sympathetic nervous system, systemic inflammatory response, impaired glucose and lipid metabolisms [5, 6]. Dyslipidemia, defined as an excessive increase in total cholesterol or triacylglycerols, with or without a concomitant decrease in high-density lipoproteins (HDL), leads to an acceleration of the atherosclerotic process in predisposed individuals and is one of the most important risk factors for vascular disease [7, 8]. Although it is not known what role OSA plays in the development of dyslipidemia, recent studies provide evidence for an independent association of intermittent hypoxia with dyslipidemia that might result in an increased risk of vascular disase in patients with OSA [9, 10].

Atherogenic subpopulations of plasma lipoproteins represent a risk factor for the development of vascular disease. Even though the concentration of atherogenic LDL and HDL subfractions in the blood is very low, they can disrupt the integrity of the vascular wall and can lead to endothelial dysfunction [11]. LDL lipoproteins are generally believed to be atherogenic lipoproteins, but the large LDL subfractions are considered to have atheroprotective properties. HDL lipoproteins are generally believed to be an atheroprotective component of plasma lipoproteins, but not all HDL subfractions exhibit this protective funtion. Some studies report potential atherogenic properties of small HDL subfractions [12, 13].

Identification and quantification of atherogenic and non-atherogenic lipoprotein subfractions in plasma is enabled by different methods (nuclear magnetic resonance, density gradient ultracentrifugation, non-denaturing gradient gel electrophoresis, the Lipoprint system). The Lipoprint system (Quantimetrix corp., Redondo Beach, CA, USA) used in this study is a polyacrylamide gel electrophoresis. It identifies 7 LDL subfractions and 10 subfractions of HDL cholesterol. LDL subfractions are classified into large (subfractions 1–2) and small, dense LDL subfractions (subfractions 3–7). HDL subfractions are classified into large subfractions (subfractions 1–3), intermediate (4–7 subfractions) and small (8–10 subfractions) [14, 15]. Small LDL3–7 and small HDL8–10 subfractions represent an atherogenic part of the lipoprotein spectrum. They are considered atherogenic because of their low recognition by the cell receptors and their ability to easily penetrate into the subendothelial space where they can form deposits of cholesterol [16]. Their high predictive value in the diagnosis of atherosclerosis-related diseases of the cardiovascular system was confirmed [17, 18].

Frequent recurrent hypoxia/reoxygenation during sleep leads to the oxidative stress which is characterized by the increased production of free radicals and lower antioxidant protection resulting in the redox imbalance [19]. Increased oxidative stress has been suggested as a potential mechanism for the development of vascular diseases in OSA patients [20]. Some studies have reported that people with OSA have increased activity of the leukocyte enzyme NADPH-oxidase producing superoxide radicals during the oxidative burst as a result of intermittent hypoxia [21, 22]. Superoxide radicals can then react with NO (vasodilator) to form a very reactive compound – peroxynitrite (ONOO^−^) [23]. This process might result in the reduction of NO bioavailability which was reported in patients with OSA [24]. In addition, RONS produced at high concentrations during intermittent hypoxia at night can damage lipids, proteins or DNA. Lipids oxidized by reactive oxygen species (ROS) can produce different oxidative products including lipid peroxides representing one of the key mechanisms of vascular damage and the increased risk of atherosclerosis [25]. Moreover, patients with OSA were found to have impaired antioxidant capacity [26]. All these results indicate a potential role of oxidative stress in the development of vascular diseases in patiens with OSA.

This study aimed to examine the presence of lipid abnormalities in patients with sleep-disordered breathing, focusing on small dense LDL and HDL subfractions. In addition, this study also focused on investigating the potential effect of oxidative stress on development of vascular diseases in OSA patients by evaluating the redox imbalance in OSA patients through monitoring the oxidative damage to lipids.

## Methods

### Study population

Only apparently healthy male subjects were enrolled. Medical records of all patients were searched for the premorbid presence of sleep apnea, vascular disease, arterial hypertension, diabetes mellitus, endocrinopathy, cancer or any other chronic diseases. Subjects were excluded if the presence of any of them was confirmed. To exclusion criteria were included also the use of any current medication or smoking.

Blood plasma samples were obtained after an overnight fast from 16 healthy male volunteers and 15 male patients of similar age with confirmed sleep-disordered breathing. The Ethical committee of the Faculty of medicine with No. 26/2021 approved this study. All project participants signed an informed consent.

### Sleep study

All subjects underwent single-night pulse oximetry monitoring with the WristOx_2_ device (model 3150, Nonin Medical, Plymouth, USA). Desaturation was defined as a drop of oxygen level > 3% with the duration of > 10 s. The desaturation index was defined as an average number of desaturations during 1 h of recording. Subjects with desaturation index < 5 were considered as a control population. Subjects with desaturation index ≥5 were considered sleep apnea patients and underwent polysomnographic assessment. Polysomnography was performed in a sleep laboratory settings using Alice 6 device (Philips-Respironics, Netherlands). Hypopnea was defined as a reduction in airflow of ≥50% for 10 s with oxygen desaturation of ≥3% or arousal and apnea as the cessation or the reduction of airflow of ≥90% for >10 s. Apnea-hypopnea index (AHI), AHI per 1 h of REM (rapid eye movement) sleep, AHI per 1 h of NREM (non-rapid eye movement) sleep, oxygen desaturation index (ODI, defined as a number of desaturations ≥3% with duration of >10 s per hour of sleep), arousal index, total sleep time and saturation of blood with oxygen were recorded.

### Plasma

Blood samples with EDTA (ethylenediaminetetraacetic acid) anticoagulant collected from individuals after 12 h fasting were spun 5 min at 1200 x g (4 °C). Collected plasma samples were stored at − 80 °C for further analyses.

### Lipid parameters

Immediately after collection of plasma samples, levels of triacylglycerols (TAG), total cholesterol (TCH), low-density lipoprotein cholesterol (LDL) and high-density lipoprotein cholesterol (HDL) were determined in the Medirex, a.s., Bratislava, Slovakia, which is a certified laboratory.

### Lipoprotein subfractions

Plasma lipoprotein subfractions were analyzed by the Lipoprint system (Quantimetrix corp., Redondo Beach, CA, USA) which is a polyacrylamide gel electrophoresis. The system can evaluate the following LDL subfractions: large LDL subfractions 1–2 which are considered atheroprotective and small dense pro-atherogenic LDL subfractions 3–7.

The Lipoprint system can also evaluate the following HDL subfractions: large HDL subfractions 1–3, which are considered anti-atherogenic and small dense HDL subfractions 8–10, which are considered pro-atherogenic. The properties of medium-sized HDL subfractions 4–7 are still under discussion [12–14].

### Lipoperoxides in plasma

Plasma lipid peroxides were determined according to El-Saadani et al. [27]. The method is based on the ability of peroxides to oxidize iodide (I^−^) to iodine(I_2_) which subsequently reacts with an excess of I^−^ to form I_3_ with the absorption maximum of 365 nm. Lipid peroxides are detected in samples spectrophotometrically (UV-1800 SHimadzu spectrophotometer).

### Statistical evaluation of results

Statistical analyses were performed by SPSS ver. 18 (SPSS Inc. Chicago, IL, USA). Results of normally distributed data are expressed as a mean ± standard deviation (SD) and of not normally distributed data as a median (lower quartile – upper quartile). To compare groups of parameters the Student’s unpaired t-test and Mann-Whitney test were used. Spearman’s correlations were performed to assess associations between variables. Univariate analysis of variance was used for a power calculation. Power was computed using α = 0.05. The *P* value < 0.05 was considered statistically significant.

## Results

This study focused on two pathomechanisms that potentially link the sleep apnea syndrome with the development of vascular diseases: dysbalance in lipid metabolism and the redox imbalance. Levels of atherogenic lipoprotein subfractions including small dense LDL and HDL subfractions were monitored in patients with OSA.

The study included 15 male OSA patients and 16 male controls. The control group included individuals of similar age, body mass index (BMI) and HDL cholesterol as the participants in the patient group. However, OSA patients had significantly higher levels of total cholesterol, LDL-cholesterol and triacylglycerols (TAG) compared to controls. Clinical characteristics of study participants are presented in Table 1.

**Table 1:** Characteristics of the study population

	Controls	Patients	***P***
**N**	16	15	
**age (years)**	35.56 ± 4.82	42.13 ± 12.21	ns
**BMI (kg/m2)**	27.46 ± 3.60	29.48 ± 4.54	ns
**TCH (mmol/L)**	4.17 ± 0.64	6.30 ± 0.98	0.006
**LDL (mmol/L)**	2.63 ± 0.62	4.01 ± 0.82	0.0001
**HDL (mmol/L)**	1.20 ± 0.29	1.21 ± 0.39	ns
**TAG (mmol/L)**	1.31 ± 0.57	1.97 ± 0.58	0.0037

Results are expressed as mean ± standard deviation (SD); *P* statistical level of significance, *N* count, *BMI* Body mass index, *TCH* Total cholesterol, *LDL* Low-density lipoproteins, *HDL* High-density lipoproteins, *TAG* Triacylglycerols, *ns* Nonsignificant

Patients with OSA had significantly higher levels of pro-atherogenic small HDL8–10 subfractions in spite of the fact that the levels of total HDL cholesterol were similar in both groups (Table 2).

**Table 2:** Plasma LDL and HDL subfractions and lipoperoxide levels in controls and patients with OSA

	Controls (***N*** = 16)	Patients (***N*** = 15)	***P***
**LDL1 (mmol/L)**	0.68 ± 0.29	0.7 ± 0.26	ns
**LDL2 (mmol/L)**	0.233 (0.155–0.388)	0.647 (0.336–0.750)	0.0003
**LDL3–7 (mmol/L)**	0 (0–0)	0.207 (0.103–0.465)	0.0003
**Large HDL1–3 (%)**	33.7 ± 15.8	17.8 ± 8.1	0.0015
**Medium HDL4–7 (%)**	22.7 ± 4.9	57.4 ± 10.4	ns
**Small HDL8–10 (%)**	13.5 (2.3–16.5)	24.32 (17.24–32.14)	0.0006
**Lipid peroxides (μmol/L)**	30.9 (22.3–44.7)	47.3 (33.9–71.3)	0.0192

Normally distributed data are expressed as mean ± standard deviation (SD), non-parametric data are expressed as median (25th – 75th percentile); *N* count, *P* statistical level of significance, *LDL* Low-density lipoproteins, *HDL* High-density lipoproteins, *ns* Nonsignificant

When comparing levels of LDL and HDL subfractions between controls and patients (Table 2) a significant elevation was found in the levels of LDL2 subfraction (*p* = 0.0003), pro-atherogenic small dense LDL3–7 (*p* = 0.0003) and small HDL8–10 subfractions (*p* = 0.0006) in patients with OSA. These patients had also significantly reduced athero-protective large HDL1–3 subfractions (*p* = 0.0015) compared to controls without OSA. The level of presumably atheroprotective large LDL1 subfraction as well as medium HDL4–7 subfractions were similar in both groups.

In addition to lipoprotein subfractions, also the the marker of oxidative damage to lipids (lipoperoxides) were examined in OSA patients. Plasma lipoperoxide levels in OSA patients were significantly higher than in healthy men (*p* = 0.0192) (Table 2).

Correlations between baseline characteristics of the sleep apnea subjects, lipid subfractions and lipoperoxides are listed in Table 3. A significant inverse correlation was found between severity of sleep apnea (expressed by AHI, ODI, time with O_2_ saturation < 90%), sleep fragmentation (expressed by arousal index) and HDL. A similar inverse correlation was found between severity of sleep apnea, sleep fragmentation and large HDL1–3. A significant inverse correlation was found also between arousal index and LDL1. AHI during REM inversely correlated with LDL2. BMI inversely correlated only with large HDL1–3.

**Table 3:** Correlations between measured parameters in patients with OSA and controls

		Age	BMI	ODI	Arousal	Time with	AHI	AHI	AHI
					index	O_2_ sat.	(REM)	(NREM)	
						< 90%			
**TCH**	r	0.248	− 0.282	−0.326	− 0.445	− 0.070	− 0.477	− 0.270	−0.270
	*P*	0.375	0.308	0.301	0.147	0.829	0.117	0.397	0.397
**LDL**	r	0.097	−0.043	− 0.147	− 0.336	0.182	− 0.364	− 0.077	− 0.077
	*P*	0.731	0.879	0.649	0.286	0.572	0.244	0.812	0.812
**HDL**	r	0.447	−0.499	**− 0.641**	− **0.627**	**− 0.578**	− 0.505	**− 0.648**	**− 0.648**
	*P*	0.095	0.058	**0.025**	**0.029**	**0.049**	0.094	**0.023**	**0.023**
**TAG**	r	−0.224	0.311	0.280	0.315	−0.126	−0.291	0.273	0.273
	*P*	0.422	0.260	0.379	0.319	0.697	0.359	0.391	0.391
**LDL1**	r	−0.243	−0.072	− 0.477	**− 0.632**	− 0.060	− 0.220	− 0.449	− 0.449
	*P*	0.382	0.800	0.117	**0.028**	0.854	0.493	0.143	0.143
**LDL2**	r	−0.278	− 0.055	−0.151	− 0.284	−0.193	**− 0.582**	−0.116	− 0.116
	*P*	0.844	0.844	0.640	0.372	0.549	**0.047**	0.721	0.721
**LDL3–7**	r	0.080	0.217	0.334	0.334	0.186	−0.123	0.383	0.383
	*P*	0.776	0.437	0.289	0.289	0.562	0.703	0.219	0.219
**Large HDL1-3**	r	0.208	**−0.572**	**−0.873**	**− 0.865**	−0.511	− 0.484	**−0.813**	**− 0.813**
	*P*	0.456	**0.026**	**0.001**	**< 0.001**	0.089	0.111	**0.001**	**0.001**
**Medium HDL4-7**	r	−0.308	0.237	0.430	0.401	0.310	0.173	0.451	0.451
	*P*	0.264	0.396	0.163	0.196	0.327	0.591	0.141	0.141
**Small HDL8-10**	r	0.095	0.261	0.259	0.280	0.133	0.098	0.217	0.217
	*P*	0.736	0.348	0.417	0.379	0.681	0.762	0.499	0.499
**Lipoperoxides**	r	−0.048	−0.175	−0.070	−0.021	−0.014	0.095	−0.007	− 0.007
	*P*	0.864	0.533	0.829	0.948	0.966	0.770	0.983	0.983

*TCH* Total cholesterol, *LDL* Low-density lipoproteins, *HDL* High-density lipoproteins, *TAG* Triacylglycerols, *BMI* Body mass index, *ODI* Oxygen desaturation index, *AHI* Apnea-hypopnea index, *REM* Rapid eye movement sleep, *NREM* Non-rapid eye movement sleep, *r* Spearman correlation coefficient, *P*- statistical level of significance. The bold text represents significant correlations

### Comparisons with other studies and what does the current work add to the existing knowledge

The results of several studies point to a higher incidence of vascular diseases in OSA patients [20]. Obstructive sleep apnea itself participates in atherogenesis through several pathomechanisms [28]. This work has focused on two of them, namely dyslipidemia and the redox imbalance represented by oxidative damage to lipids. In patients with OSA and healthy volunteers plasma lipid profile and lipoperoxide levels were compared. Both groups had comparable age and BMI but individuals with OSA showed significantly elevated total cholesterol, LDL cholesterol and triacylglycerol levels. Our results are consistent with some other studies that have shown elevated levels of total cholesterol, LDL cholesterol and triacylglycerols in individuals with OSA [29–32]. HDL cholesterol levels were comparable in both groups.

In this study, despite comparable HDL cholesterol levels, significantly higher levels of presumably atherogenic small HDL subfractions were found in patients with OSA, as well as significantly lower levels of large, atheroprotective HDL subfractions. This finding suggests a pro-atherogenic lipid profile in patients with OSA. In contrast to this study, where similar HDL cholesterol levels in both groups were found, some studies report decreased HDL cholesterol levels [32–34] or increased HDL cholesterol levels in individuals with OSA [28].

A subanalysis of OSA patients in the current study revealed an inverse correlation between the severity of sleep apnea, sleep fragmentation and HDL. A similar inverse correlation appeared between sleep apnea severity, sleep fragmentation and „atheroprotective “large HDL1–3. A significant inverse correlation was found also between arousal index and „atheroprotective “LDL1. Another „atheroprotective “LDL subfraction – LDL2 inversely correlates with AHI during REM sleep. These findings suggest a dose-dependent inverse association between the sleep apnea severity, sleep fragmentation and „atheroprotective “LDL and HDL subfractions. In contrast to previous findings, respiratory events during NREM sleep might play a more important role in changes of lipoprotein levels when compared to respiratory events during REM sleep [35].

This study is the first one to report increased levels of „atherogenic “small HDL8–10 subfractions and reduced levels of „atheroprotective “large HDL1–3 subfractions in people with OSA compared to the participants in the control group. Preliminary results indicate changed proportion of large and small subfractions of HDL cholesterol in OSA patients towards a proatherogenic HDL profile, so far without a change in the concentration of the total HDL cholesterol. A similar pattern can be observed in LDL subfractions. Individuals with OSA and controls have similar levels of large atheroprotective LDL1 subfraction, but OSA patients have significantly increased levels of small dense LDL3–7 subfractions, indicating a lipoprotein proatherogenic phenotype. This might be one of the first consequences of intermittent hypoxia and sleep fragmentation on the development of dyslipidemia.

Despite the fact that exact mechanisms linking sleep apnea with the decrease of „atheroprotective “and increase of „atherogenic “lipoprotein subfractions remain unknown, oxidative stress belongs to the known mechanisms leading to the HDL dysfunction in OSA patients [36]. This was one of the reasons for the redox imbalance assessment in the current study. It is assumed that markers of oxidative stress may be increased due to repeated short cycles of intermittent hypoxia followed by reoxygenation, during which free radicals are formed causing oxidative damage to biomacromolecules and disrupting vascular homeostasis [37]. These processes may result in increased cardiovascular and cerebrovascular risk in individuals with sleep-disordered breathing [38, 39]. Significantly increased levels of lipoperoxides in plasma were found in individuals with OSA compared to the control group. Similarly, Hopps el al [40]. found increased lipoperoxide levels in patients with serious OSA. Our results are in agreement with other studies determining elevated levels of other markers of oxidative damage to lipids such as thiobarbituric acid reactive substances in plasma [41, 42] or 8-isoprostane in exhaled breath condensate of OSA patients [43]. However, some studies are reporting unchanged plasma levels of thiobarbituric acid reactive substances compared to healthy individuals [25]. In addition, also elevated levels of markers of oxidative damage to proteins [44] and nucleic acids [45] have been repeatedly reported in individuals with OSA. Taken together, the data described above indicate that redox imbalance might participate in the atherogenic process of individuals with OSA [46].

### Study strength and limitations

This study is the first one to report elevated levels of „atherogenic “small HDL8–10 subfractions and reduced levels of „atheroprotective “large HDL1–3 subfractions in OSA subjects. Results from the subpopulation with OSA suggest a dose-dependent inverse association between the sleep apnea severity, sleep fragmentation and „atheroprotective “LDL and HDL subfractions. Additionally, the redox imbalance – another pathomechanism contributing to the atherogenic process, was confirmed in individuals with OSA.

Enrollment of the healthy population is another strength of this study. The selection of subjects without any known vascular risk factors could elucidate the effect of intermittent hypoxia and sleep fragmentation on lipoprotein levels and redox balance. However, healthy subjects were selected only according to the detailed search of the medical records. Additional use of functional, laboratory, paraclinical tests or imaging could be beneficial to avoid the presence of patients with asymptomatic vascular disease in a study population. Measurement of subclinical atherosclerosis (e.g. thickness of carotid intima-media, endothelial dysfuntion) could be also beneficial to exclude subjects with the pre-existing atherosclerotic process [47, 48]. It could also elucidate links between the redox imbalance, lipoprotein abnormalities and subclinical atherosclerosis in OSA subjects.

A small sample size is one of the main limitations of this study. However, the analyses show adequate power to reveal differences in TCH (power = 0.961, *P* = 0.001), LDL (power = 0.999, *P* < 0.001), TAG (power = 0.862, *P* = 0.004), LDL2 (power = 0.948, *P* = 0.001), LDL3–7 (power = 0.943, *P* = 0.001), large HDL1–3 (power = 0.925, *P* = 0.001), small HDL8–10 (power = 0.936, *P* = 0.001). On the other hand, the study could be underpowered to reveal differences in lipoperoxides (power = 0.551, *P* = 0.039). Therefore, there is a need for larger longitudinal trials confirming the effect of OSA on dyslipidemia and the redox imbalance. Future studies should also elucidate mechanisms linking sleep apnea with the decrease of „atheroprotective “lipoprotein subfractions. It is necessary to admit, that lifestyle interventions (including diet and physical activity) influence levels of lipoprotein subfractions [49]. Additional limitation of this study is the absence of detailed biometry, diet and physical activity.

## Conclusion

Abnormalities in lipoprotein levels in OSA patients and the redox imbalance can lead to an acceleration of the atherogenic proces in predisposed individuals and thus represent a risk factor for the development of vascular disease in these patients. Preliminary results of the current study describing abnormalities in lipoprotein levels in patients with OSA are consistent with the hypothesis of a frequent occurrence of lipid metabolism disorders in individuals with OSA. In the future studies it could be interesting to examine some novel hypotheses suggesting a link between intermittent hypoxia and composition of gut microbiota [50] or hypothesis on synergistic relationship between insulin and leptin [51] or impact of intermittent hypoxia on glucose metabolims. In order to find new therapeutic targets for OSA it is important to understand its ethiology.

## Data Availability

The datasets used and/or analyzed during the current study are available from the corresponding author on reasonable request.

## Abbreviations

BMI: Body mass index
HDL: High-density lipoprotein cholesterol
IDL: Intermediate-density lipoproteins
LDL: Low-density lipoprotein cholesterol
OSA: Obstructive sleep apnea
TAG: Triacylglycerols
TCH: Total cholesterol
REM: Rapid eye movement sleep
NREM: Non-rapid eye movement sleep
